# Supplementary material for: Emulative, coherent, and causal dynamics between large-scale brain networks are neurobiomarkers of Accelerated Cognitive Ageing in epilepsy
Source: PLoS One. 2021 Apr 16;16(4):e0250222. doi: 10.1371/journal.pone.0250222 (PMC8051821; doi:10.1371/journal.pone.0250222)
Supplement: S1 File — (PDF) [file pone.0250222.s001.pdf]

SUPPORTING INFORMATION

BEST STATIC FC-, GC-, AND EGN-BASED FEATURES SELECTED FOR CLASSIFICATION OF THE ACA / HEALTHY PARTICIPANTS.

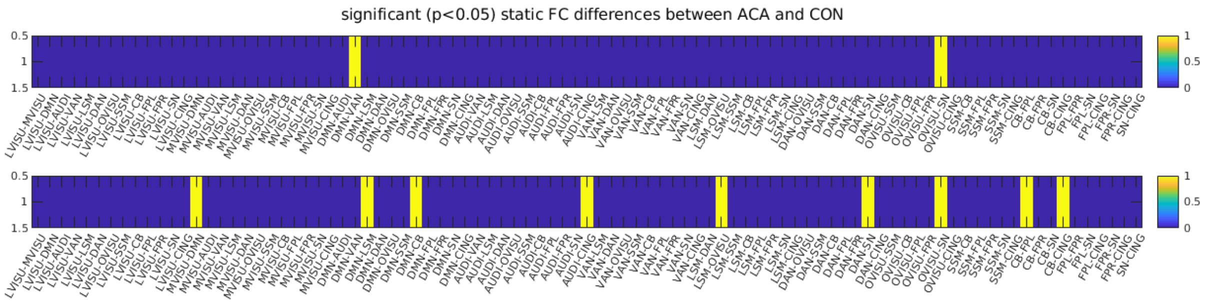

S1 Fig. Significant differences in static functional connectivity between ACA and controls. Top – RS1; bottom – RS2; yellow – significant (p<0.05) features; dark blue – non-significant features.

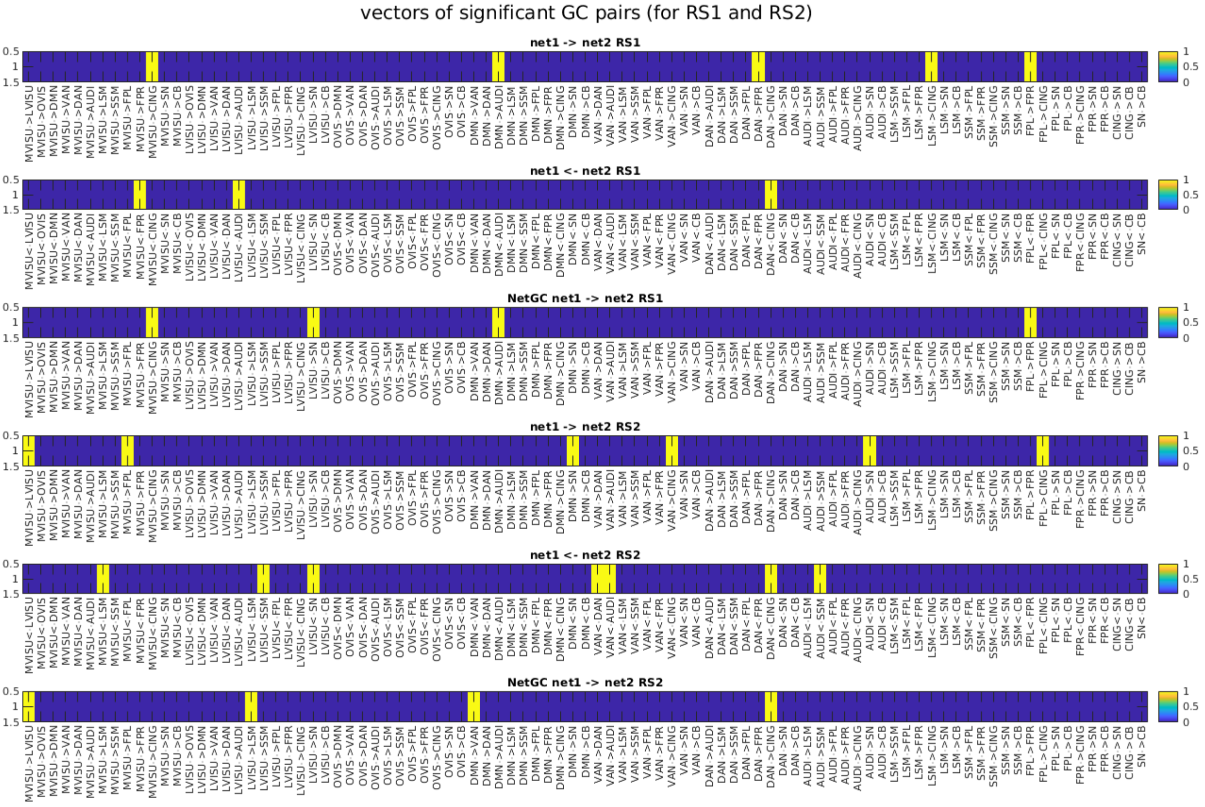

S2 Fig. Significant GC differences between ACA and controls. -> – causal direction; net1(net2) – networks; NetGC – (net1->net2 - net1<-net2); yellow – significant (p<0.05) features; dark blue – non-significant features.

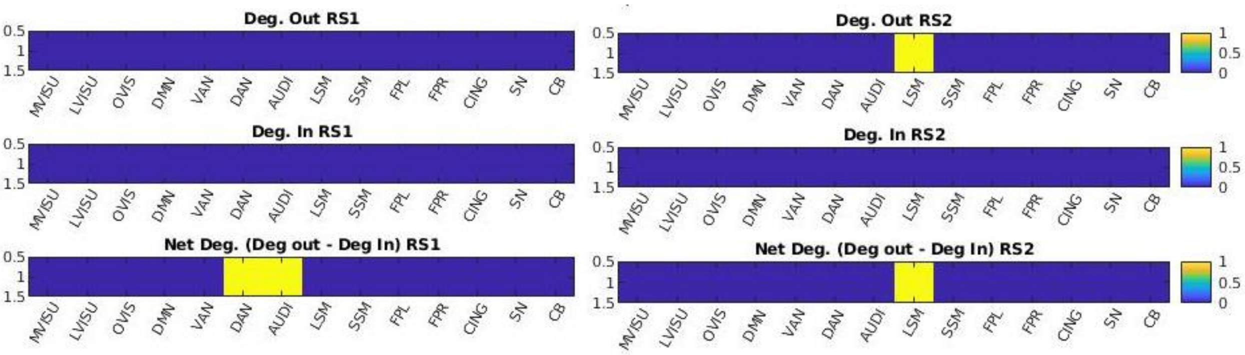

S3 Fig. Significant difference between ACA and controls in the GC degrees of the networks. Top – GC deg. In; middle – GC deg. out; bottom – Net GC deg. (deg. out - deg. in); left – RS1; right – RS2. yellow – significant ($p<0.05$) features; dark blue – non-significant features.

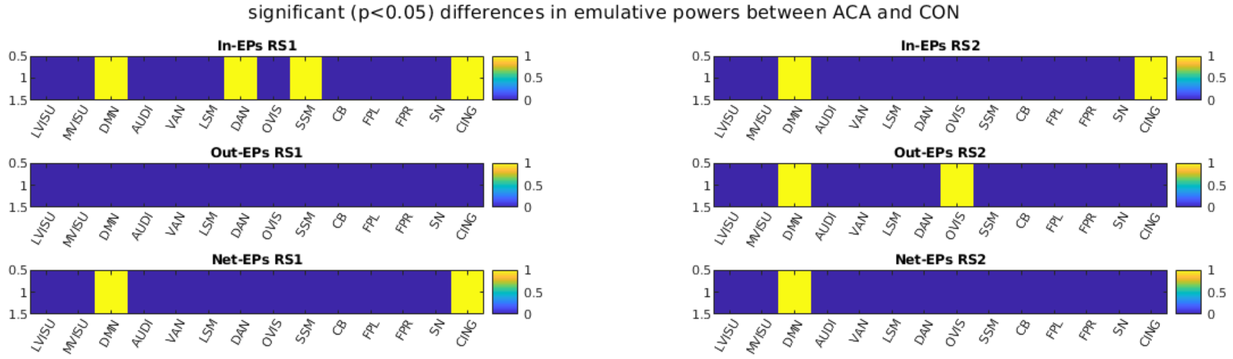

S4 Fig. Significant differences between ACA and controls in network emulative powers (Eps). Top – the In-Eps; middle – Out-Eps; bottom – Net-EPs. Net-EPs = Out-EPs - In-EPs. left – RS1; right – RS2; yellow – significant ($p<0.05$) features; dark blue – non-significant features.
