# Supplementary figures and images for: Emulative, coherent, and causal dynamics between large-scale brain networks are neurobiomarkers of Accelerated Cognitive Ageing in epilepsy
Source: PLoS One. 2021 Apr 16;16(4):e0250222. doi: 10.1371/journal.pone.0250222 (PMC8051821; doi:10.1371/journal.pone.0250222)

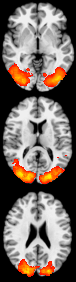

Supplement: S1 Data — (ZIP) [file pone.0250222.s002.zip › Data/18_Networks/0000.png]

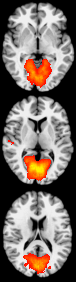

Supplement: S1 Data — (ZIP) [file pone.0250222.s002.zip › Data/18_Networks/0001.png]

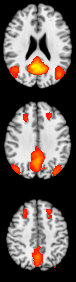

Supplement: S1 Data — (ZIP) [file pone.0250222.s002.zip › Data/18_Networks/0002.png]

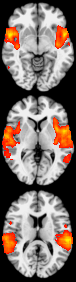

Supplement: S1 Data — (ZIP) [file pone.0250222.s002.zip › Data/18_Networks/0003.png]

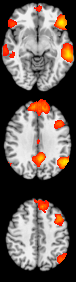

Supplement: S1 Data — (ZIP) [file pone.0250222.s002.zip › Data/18_Networks/0004.png]

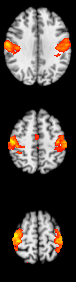

Supplement: S1 Data — (ZIP) [file pone.0250222.s002.zip › Data/18_Networks/0005.png]

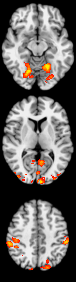

Supplement: S1 Data — (ZIP) [file pone.0250222.s002.zip › Data/18_Networks/0006.png]

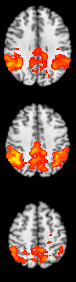

Supplement: S1 Data — (ZIP) [file pone.0250222.s002.zip › Data/18_Networks/0007.png]

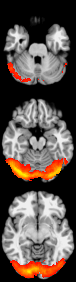

Supplement: S1 Data — (ZIP) [file pone.0250222.s002.zip › Data/18_Networks/0008.png]

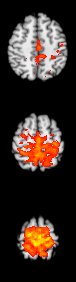

Supplement: S1 Data — (ZIP) [file pone.0250222.s002.zip › Data/18_Networks/0009.png]

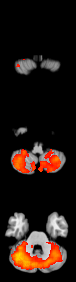

Supplement: S1 Data — (ZIP) [file pone.0250222.s002.zip › Data/18_Networks/0010.png]

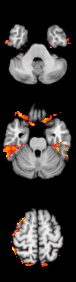

Supplement: S1 Data — (ZIP) [file pone.0250222.s002.zip › Data/18_Networks/0011.png]

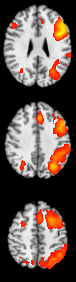

Supplement: S1 Data — (ZIP) [file pone.0250222.s002.zip › Data/18_Networks/0012.png]

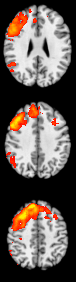

Supplement: S1 Data — (ZIP) [file pone.0250222.s002.zip › Data/18_Networks/0013.png]

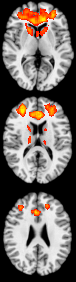

Supplement: S1 Data — (ZIP) [file pone.0250222.s002.zip › Data/18_Networks/0014.png]

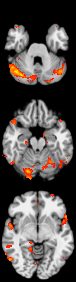

Supplement: S1 Data — (ZIP) [file pone.0250222.s002.zip › Data/18_Networks/0015.png]

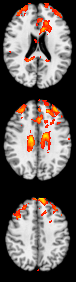

Supplement: S1 Data — (ZIP) [file pone.0250222.s002.zip › Data/18_Networks/0016.png]

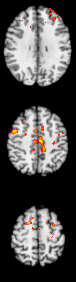

Supplement: S1 Data — (ZIP) [file pone.0250222.s002.zip › Data/18_Networks/0017.png]
