# Supplementary material for: Emulative, coherent, and causal dynamics between large-scale brain networks are neurobiomarkers of Accelerated Cognitive Ageing in epilepsy
Source: PLoS One. 2021 Apr 16;16(4):e0250222. doi: 10.1371/journal.pone.0250222 (PMC8051821; doi:10.1371/journal.pone.0250222)
Supplement: Attachment — Submitted filename: Response_to_Reviewers.docx [file pone.0250222.s003.docx]

Dear Editor, dear Reviewers,

We would like to thank you for the interest you have shown in our manuscript, and for your insightful and constructive comments. We have carefully considered and addressed each of the comments.

Please find below the detailed answers to your comments (in dark: your comments; in blue: our responses/changes).

Sincerely,

Antoine and all co-authors

****

Editor:

Journal Requirements:

When submitting your revision, we need you to address these additional requirements.

1. Please ensure that your manuscript meets PLOS ONE's style requirements, including those for file naming. The PLOS ONE style templates can be found at

https://journals.plos.org/plosone/s/file?id=wjVg/PLOSOne_formatting_sample_main_body.pdf and

https://journals.plos.org/plosone/s/file?id=ba62/PLOSOne_formatting_sample_title_authors_affiliations.pdf

We ensured the manuscript meets PLOS ONE’s style requirements.

2.  In your Data Availability statement, you have not specified where the minimal data set underlying the results described in your manuscript can be found. PLOS defines a study's minimal data set as the underlying data used to reach the conclusions drawn in the manuscript and any additional data required to replicate the reported study findings in their entirety. All PLOS journals require that the minimal data set be made fully available. For more information about our data policy, please see http://journals.plos.org/plosone/s/data-availability.

Yes, we now make the minimal data set available. The raw data (fMRI) are subject to privacy protection and are not publicly available.

Upon re-submitting your revised manuscript, please upload your study’s minimal underlying data set as either Supporting Information files or to a stable, public repository and include the relevant URLs, DOIs, or accession numbers within your revised cover letter. For a list of acceptable repositories, please see http://journals.plos.org/plosone/s/data-availability#loc-recommended-repositories. Any potentially identifying patient information must be fully anonymized.

The study’s minimal data set has been added to Supporting Information files (Data.zip).

Important: If there are ethical or legal restrictions to sharing your data publicly, please explain these restrictions in detail. Please see our guidelines for more information on what we consider unacceptable restrictions to publicly sharing data: http://journals.plos.org/plosone/s/data-availability#loc-unacceptable-data-access-restrictions. Note that it is not acceptable for the authors to be the sole named individuals responsible for ensuring data access.

We have restriction in sharing the raw fMRI data publicly, this is explained more in detail in the Data Availability statement.

We will update your Data Availability statement to reflect the information you provide in your cover letter.

3. Please include captions for your Supporting Information files at the end of your manuscript, and update any in-text citations to match accordingly. Please see our Supporting Information guidelines for more information: [http://journals.plos.org/plosone/s/supporting-information](about:blank).

We have added the dataset file in Supporting Information, added the captions, and updated the in-text citations.

****

Reviewer #1:

Overall, the study explores the biomarkers from the functional connectivity perspective among ACA in a scientifically sound method. The study has novelty in exploring the interactions between compound networks in the brain particularly in ACA subjects.

There are a few constructive criticisms to help improve the manuscript, which are as follows:

the ABSTRACT section only include one paragraph and don’t use any abbreviations.

We made it one paragraph, and we removed the abbreviations, except for ACA since it is introduced in the first line, and used this abbreviation many time in the abstract.

Original text: grey highlight = accuracy values; yellow highlight = best averages accuracy in each set-up; blue highlights = accuracy > 80%.
Suggestion: Do not highlights any words other than the numbers in the tables of the text.

We removed the highlighting in the caption.

Original text: Table 3 and Table 4
Suggest to attach some graphs to depict the results of Table 3 and Table 4 intuitively.

We added a new figure to grasp the results of Tables 3 and 4 more intuitively: Fig 6. with the caption: “Graphical summary of the classification results. A: best performances of each network connectivity metric. B: best performances of each classifier algorithm.” (Section 4.2)

We also introduce the figure with the sentence: “For a more intuitive visualization of the results presented above, we graphically summarize Tables 3 and 4 in Figs 6A and 6B respectively.” at the end of Section 4.2.

Many abbreviations of terms were used in the manuscript. It is suggested that check them all, ensure they were abbreviated on the first time and use their abbreviations in the rest of manuscript.

We have addressed this issue.

On the other hand, although it is allowed that using brackets to supplement or explain the main text, but there are too many of them in this manuscript. It is suggested that delete some dispensable contents in brackets or write them in the sentence directly.

Yes, we did remove dispensable parentheses as in:

- “[…] in a bigger dataset stratified, e.g. through duration since onset of epilepsy or age ranges, could even help predicting […]”. (Section 2)
- “, RS1 and RS2,”; “, among all participant/scans,”; “, i.e. cognitive decline,”. (Section 3.1)
- “leading to two times 24 […]”; “namely the In-EPs, Out-EPs, and Net-EPs,”; “by the other networks”. (Section 3.2)

- “(ii) features that involved the DMN, DAN or SN, since these are networks often mentioned in ageing and cognitive decline literature.”. (Section 3.3)
- “An example of a pairwise in-phase time-of-coherence […]”; “similar to the increase of in-phase coherence in ACA patients”. (Section 5.1)
- “In general, for RS1 and RS2, the DMN In-EPs of the patients were negative […]”; “And following the evolution of such dynamic or static fMRI-based brain features”; “or to assess lasting effects of different treatments of psychiatric disorders”. (Section 5.2)
- “[…] that the accuracy of SVM classification gets simply more reliable, with less variability in performances, as the training sample size increases”; “Nevertheless, the cross-center robustness still needs to be tested.”. (Section 5.3)

Suggest the manuscript should be smoothed in English writing.
Such as “ An important drawback of this classification technique is that it usually needs a large amount of data to be trained upon; plus, the understanding and explanation of the feature maps obtained at different CNN layers can be arduous and troublesome.”, the word ‘plus’ should be replaced by a more formal one.
“Finally, evaluation of brain dynamics can be done through an evolutionary game theory on networks (EGN) approach.”
Suggestion: Finally, evaluation of brain dynamics can be done through an evolutionary game theory (EGN) approach on brain networks.

We have addressed these issues and changed the text as suggested (in Sections 2 and 3.2).

“Figure 4 depicts the neurodynamics features extraction of the 4 approaches. Those features were extracted for each RS session.”
Suggest to cite the Figure 4 in the main body of the manuscript rather than put it as an independent paragraph. Besides, it is suggested that add serial annotations on different part in Figure 4 and cite them in the main text of 3.2 section.

We removed the sentences “Figure 4 depicts the neurodynamics features extraction of the 4 approaches. Those features were extracted for each RS session.” at the end of Section 3.2. We also added A, B, C, and D annotations in Fig 4, and we now cite Fig 4A, Fig 4B, Fig 4C, Fig 4D, in the main text of Section 3.2.

The caption of Fig 4 has also changed accordingly: “Fig 4. Network feature extraction and statistical analysis. A: extraction of the static functional connectivity features. B: computation of the Granger causality matrices and vectors. C: Emulative power vectors derived from the EGN method. D: Wavelet coherence approach and the time-of-coherence metric extraction.”

****

Reviewer #2:

This paper studied the classification between ACA in epilepsy and normal people, using the machine learning method and functional connectivity matric from fMRI. The authors constructed four kinds of functional matric, and then extracted the significant features that were put into the four classifiers to divide the ACA and control subjects. This topic combining brain networks and machine learning methods is the frontier in network neuroscience. This work is well organized, the methods are clear and the results looks like confident. I will recommend the paper published in plos one after a minor revision.

1. This work actually used four kinds of functional matric, i.e., sFC, GC, EGN and WCoh. The sFC, GC and EGN are actually static matric, and WCoh is dynamics. So I suggest that the ‘dynamic features’ (such as below the Figure. 2 description) should be revised to network features or some things.

We think that GC and EGN are metrics describing dynamics in the brain, therefore we use the term neurodynamics, or dynamics features/metrics throughout the manuscript. However, we agree that when we talk about all 4 metrics (including static FC), the use of another term such as ‘network features’ is preferable. Hence, we changed terms as follows:

- “The time series associated with these 14 RSNs were then used to extract our brain ~~dynamics~~ networks features as explained in the next section” (Section 3.1)
- “Fig 4. - ~~Dynamics~~ Network feature extraction and statistical analysis.”; “Finally, we tested classifiers with combined network features […]”; “[…] was performed in the classification analyses per network connectivity metric” “[…] “combining all features selected by the statistical analyses of all static and dynamics metrics. In the combined network features setups, a dozen (through refinements) classifiers were also tested.” “only the best classifiers performances per feature type” (Section 3.2)
- “4.1	SELECTION OF THE BEST ~~DYNAMICS~~ NETWORK FEATURES”, (title, Section 4.1)
- “we obtained the best descriptors (features) for each ~~dynamics~~ metric”; “In Table 3, the best performances of each individual feature type are shown.”; “Table 3 - Summary tables of the best classifier performances for each network connectivity measures: EGN, Coherence, sFC, and GC.“; “After assessing the best features of each ~~dynamics~~ metric […]”. (Section 4.2)

Meanwhile, in the title and abstract (second paragraph), there is no description about sFC.

sFC was indeed not introduced in the abstract. We addressed this issue by adding the sentence: “We also calculated the widely used static functional connectivity to compare the methods.” in Abstract section.

2. In the abstract, the SVM should be given the full name.

Yes, it should. We made the change.

3. Sec. 3.3, NEP is net-EP? I also noticed that the permutations tests were performed to detect the significant difference of network features between two groups. Through the paper, I think that this test should detect the significant features within each group, because the comparison between ACA and control groups is performed at Sec. 4.1.

NEP was standing for Network EP (i.e. In-EP, Out-EP, and Net-EP), but since we never introduced that abbreviation before in the text, it was confusing. We therefore changed ‘NEP’ to simply ‘EP’.

Regarding the permutation testing, it is indeed performed between the two groups. In Section 3.3 we describe the permutation testing, whereas in Section 4.1, we explain that the results can be found in Supporting Information.

4. In fig5, ‘s’ is missed for the x-labels, and what does it mean for three ‘+’.

We have removed the ‘s’ for seconds, since we already wrote ‘periods (in s)’. We also added a short explanation for the ‘+’ in the Fig 5 caption.

5. Sec4.2, ‘Note that ….. the classification accuracy when added’, can you explain why? The important idea of this paper is to detect significant features that are supposed to be better for the classification, if the not-best features can also improve the accuracy, does it mean that the ‘significant features’ are actually not significant?

In case of the EGN-based classifiers, we decided to add more (non-significant) features that involved the DAN, DMN, and SN, which was part of our classifier refinement process, as mentioned in Section 3.3: “[…] (ii) features that involved the DMN, DAN or SN, since these networks are often mentioned in ageing and cognitive decline literature.”

To respond to why adding the Out- and Net-EPs did improved classification, we wrote a short explanation in section 4.2, as follows:

“This phenomenon can be explained by the fact that, even though Out- and Net-EPs of the DAN are not statistically significant (p>0.05), the way they are pooled with their significant counterparts (In-EPs) in SVM and LDA algorithms, can allow classifiers to be more discriminative.”

Overall, this does not mean that the ‘significant features’ are not significant, but rather that some of the (statistically) non-significant features can still be relevant and help classifiers perform better.

6. In table3, I noticed that the term of WCon mixing Loocv_RS2 has 100% accuracy. If this accuracy perfectly classifies the ACA and control subjects, why used another methods and obtained an average accuracy. That is, why using five methods for validation should be explained.

We already explain shortly why we preferred not to trust fully the 100% accuracy in Section 4.2:

“It is worth noting that in some set-ups DT algorithm reached 100% accuracy, such as for the LOOCV of RS2 with the mix of 6 features (Table 4); but this was not consistent, since in the same set-up the RS1-trained-and-RS2-validated classifier reached only 66.7% accuracy.”

With regard to why we used five methods for validation, this is specifically to avoid to have these ‘chance’ results (e.g. 100% accuracy) and to assess consistency, since the sample size is relatively small. We added a sentence to make it explicit in the text (Section 3.3): “5 validation methods are used to allow us to fully trust the performances of the classifiers. It is also more robust: we can assess consistency and avoid depicting unreproducible ‘chance’ performances.”

7. Indeed, the GC measures the direct connectivity between regions, the papers ‘Spontaneous electromagnetic induction promotes the formation of economical neuronal network structure via self-organization’ and ‘Investigating driver fatigue versus alertness using the granger causality’ may be helpful for detecting the deeper significant features.

These papers are interesting. We could surely dive more into Granger causality, and make deeper analysis to extract relevant brain features to train and validate new classifiers. However, these deeper features must be extracted from EEG, which is out of the scope of this paper (which looks at between-network features extracted with fMRI).
